# Multi-Objective Optimization of Braun-Type Exothermic Reactor for Ammonia Synthesis

**DOI:** 10.3390/e24010052

**Published:** 2021-12-28

**Authors:** Tianchao Xie, Shaojun Xia, Chao Wang

**Affiliations:** College of Power Engineering, Naval University of Engineering, Wuhan 430033, China; archiboldxie@163.com (T.X.); Victoria329@163.com (C.W.)

**Keywords:** ammonia synthesis, exothermic rate, entropy generation rate, finite-time thermodynamics, multi-objective optimization

## Abstract

The exothermic reactor for ammonia synthesis is a primary device determining the performance of the energy storage system. The Braun-type ammonia synthesis reactor is used as the exothermic reactor to improve the heat release rate. Due to the entirely different usage scenarios and design objectives, its parameters need to be redesigned and optimized. Based on finite-time thermodynamics, a one-dimensional model is established to analyze the effects of inlet gas molar flow rate, hydrogen–nitrogen ratio, reactor length and inlet temperature on the total entropy generation rate and the total exothermic rate of the reactor. It’s found that the total exothermic rate mainly depends on the inlet molar flow rate. Furthermore, considering the minimum total entropy generation rate and maximum total exothermic rate, the NSGA-II algorithm is applied to optimize seven reactor parameters including the inlet molar flow rate, lengths and temperatures of the three reactors. Lastly, the optimized reactor is obtained from the Pareto front using three fuzzy decision methods and deviation index. Compared with the reference reactor, the total exothermic rate of the optimized reactor is improved by 12.6% while the total entropy generation rate is reduced by 3.4%. The results in this paper can provide some guidance for the optimal design and application of exothermic reactors in practical engineering.

## 1. Introduction

As solar illumination fluctuates greatly with weather conditions, the thermal storage system is pivotal for the stable operation of solar thermal power generation systems. The industrialization of solar thermal power systems equipped with thermal storage systems will effectively improve the global energy supply structure and promote emission peaking and carbon neutrality. Luzzi et al. [1,2], Lovegrove et al. [3,4] and Kreetz et al. [5,6] from the Australian National University developed the first paraboloidal dish solar thermal power pilot plant equipped with an ammonia-based thermochemical energy storage system, which realized continuous power generation through a whole day. The ammonia synthesis exothermic reactor is a critical component determining the power generation performance of thermal storage systems. Consequently, the analysis and optimization of the exothermic reactor will promote the power generation efficiency of the thermal storage system.

Existing studies of exothermic reactors for ammonia synthesis have mainly concentrated on single-tube reactors because their operation and design parameters are more easily controlled. Long and Liao [7] established a single-tube filled-bed ammonia synthesis reactor model and pointed out that the maximum heat output could be achieved when the reaction temperature was 850 °C and the maximum exergy output was at a reaction temperature of 650 °C. Chen et al. [8,9] designed a tubular ammonia synthesis system that could heat 26 MPa supercritical steam to 650 °C. The optimization was carried out for the minimum volume of wall material, and it was found that the ammonia synthesis reactor with a smaller tube diameter could effectively enhance heat transfer. Abdiwe et al. [10] analyzed the performances of ammonia decomposition and synthesis reactors in a closed-loop system, and found that maintaining the optimum mass flow rate was the key factor to achieve the maximum exothermic rate of the system. Flórez-Orrego et al. [11,12] applied the methane steam reforming process to ammonia synthesis, performed the exergy analysis of the entire ammonia synthesis process, and obtained an overall exergy efficiency of 66.36%. Ksasy et al. [13] optimized the length of a tubular autothermal ammonia synthesis reactor for the highest reactor profit. Babu et al. [14] optimized the length of the reactor for the lowest cost.

Finite Time Thermodynamics [15,16,17,18] (FTT) has made significant research progress in many kinds of thermodynamic devices, processes and cycles [19,20,21,22,23,24,25,26,27,28,29] since its inception in the 1970s. In chemical engineering, Nummedal et al. [30] found that reducing the length of the ammonia synthesis reactor or increasing reaction heat released could reduce the total entropy generation rate of the reactor effectively. Maansson and Andresen [31] calculated the optimum axial temperature distribution of the ammonia synthesis reactor with the objective of the highest ammonia yield at the same inlet conditions. Badescu et al. [32] obtained the optimal axial temperature distribution, pipe diameter and catalyst particle size distribution of the ammonia decomposition reactor by using the minimum heat flux required to achieve the predetermined ammonia decomposition rate and the maximum ammonia decomposition rate as the optimization objectives, respectively. Koeijer et al. [33] optimized the height of four catalytic beds of the sulfur dioxide oxidation reactor and the temperature difference of five heat exchangers and obtained a 16.7% reduction in the total entropy generation rate. Kong et al. [34,35] optimized the heat source temperature distribution of the hydrogen iodide decomposition reactor for the minimum entropy generation rate. Li et al. [36,37] optimized the inlet parameters of a steam methane reforming reactor heated by molten salt and got a 22% reduction in the total entropy generation rate.

However, the existing studies of ammonia synthesis exothermic reactors mainly focus on single-tube reactors, with the maximum exothermic rate as well as the minimum exergy destruction rate as their analysis and optimization objectives [8,9,10,11,12,13,14,15]. Due to the small diameter, the exothermic rate is generally about 1–4 kW, which does not fully meet the demand for power generation measured at a megawatt or even ten-megawatt level. To improve the total exothermic rate and promote the engineering practice of the heat storage system using the technology accumulation of industrial ammonia synthesis, the industrial Braun-type ammonia synthesis reactor is introduced into the heat storage and power generation system as the exothermic reactor for ammonia synthesis.

Braun ammonia synthesis reactors usually consist of two to four adiabatic reactor towers with one heat exchanger behind each tower. Industrial reactors are designed to save energy and increase ammonia production and outlet ammonia content, so their design and operating parameters are not fully applicable to thermal storage and power generation scenarios. Therefore, this paper applies finite-time thermodynamics to establish a one-dimensional model to analyze the influence of inlet gas molar flow rate, hydrogen to nitrogen ratio, length and inlet temperature of individual reactors on the entropy generation rate and the total exothermic rate. And finally, taking inlet flow rate, lengths and temperatures of three reactor towers as optimization variables, the NSGA-II algorithm is carried out for the multi-objective optimization of the minimum entropy generation rate and the maximum total exothermic rate.

## 2. Physical Model of Ammonia Synthesis Exothermic System

**Figure 1:**
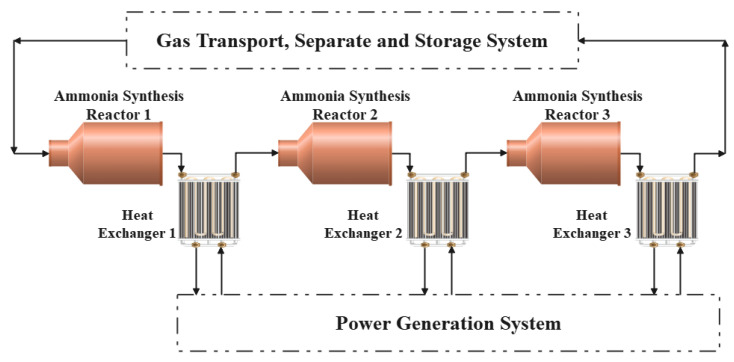
Braun-type three tower ammonia synthesis exothermic system.

Braun three-tower ammonia synthesis system is composed of three adiabatic synthesis towers equipped with one heat exchanger for cooling behind each synthesis tower. The feed gas flows in from one end of the reactor, and the synthesis reaction occurs and exotherms under the action of the catalyst, causing the temperature of the gas mixture to increase. Then, it flows into the tube pass of the counter-current heat exchanger behind the reactor and heats the water vapor in the shell pass. Then, the cooled gas mixture flows into the next reactor, and so on. What should be noted is that there is no mass transfer between system and outside during the process as the Braun synthesis tower is cooled by heat exchangers rather than quench. In other words, there is no new reactants are added and no ammonia is separated between the inlet of reactor 1 and the outlet of exchanger 3. The post-reaction gas mixture from heat exchanger 3 will flow into the gas transport, separate and storage system, where the separation and storage of the gas mixture will take place to allow the energy storage system to operate in a cyclic manner. However, this gas transport, separate and storage system is not within the scope of this paper. Figure 1 displays the system chart about the ammonia synthesis exothermic reactor.

### 2.1. Physical Model of Ammonia Synthesis Reactors

**Figure 2:**
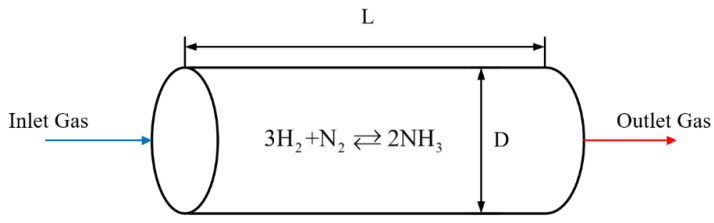
Tubular filled-bed ammonia synthesis reactor.

This paper uses a one-dimensional model, and the ammonia synthesis reactor tower can be simplified to a tubular filled-bed reactor with a large diameter. The gas is thoroughly mixed uniformly in the radial direction, the radial temperature difference inside the reactor and heat exchanger is ignored, and no back-mixing occurs during the axial flow. A model diagram of a tubular filled-bed ammonia synthesis reactor is shown in Figure 2, and its relevant parameters are listed in Table 1.

Because the reaction pressure of the ammonia synthesis process reaches 15 MPa and the temperature can achieve 600–800 K, the ideal gas equation of state has a large error. So, all the property parameters involved, such as the specific heat capacity at constant pressure, thermal conductivity, density, and kinetic viscosity are calculated by the REFPROP software developed by NIST.

#### 2.1.1. Reaction Kinetic Equations

The reaction kinetic model for ammonia synthesis obeys the Temkin–Pyzhev reaction rate equation. For real gases, directly calculate the partial pressures of the components by molar fraction is not accurate, so, to keep the equation form simple, activity is introduced into the reaction rate equation [31]:

(1) $$r=\frac{K_{b}}{{K_{c}}^{2\alpha }}\left[{K_{a}}^{2}\alpha_{N}{\left(\frac{{\alpha_{H}}^{3}}{{\alpha_{Z}}^{2}}\right)}^{\beta }-{\left(\frac{{\alpha_{Z}}^{2}}{{\alpha_{H}}^{3}}\right)}^{1-\beta }\right]$$

where, r is the ammonia synthesis reaction rate in mol/kg·s; $\alpha_{i}$ is the partical pressure calculated by the activity coefficient of corresponding substances (i=H, N, Z and A, which correspond to hydrogen, nitrogen, ammonia and argon, respectively) in bar; β is a constant determined by the state of nitrogen decomposition on the catalyst surface which is taken as 0.75 in this paper [31].

The $K_{a}$ in Equation (1) is the equilibrium constant of the ammonia synthesis reaction, which is given by the following equations [31]:

(2) log(KaKa*)=[0.1191849T−1+25122730T−4+38.76816T−2∑ixiAi12+64.49429T−2(∑ixiAi12)2]p

(3) $$\begin{array}{ll} \log{K_{a}}^{*}= & -2.691122\log T-5.519265\times 10^{-5}T+2.6899\\ & +1.848863\times 10^{-7}T^{2}+2001.6T^{-1} \end{array}$$

where *T* is the reaction temperature in *K*; $x_{i}$ is the molar fraction of the component *i*; $A_{i}$ is the parameter in Beattie–Bridgeman equation of state for the pure gas of the component *i*, which can be found in Table 2 [31].

$K_{b}$ and $K_{c}$ in Equation (1) essentially characterize the reaction rate constants, which are calculated by [31]:

(4) $$K_{b}(T)=K_{b0}\exp(-\frac{E_{b}}{RT})$$

(5) $$K_{c}(T)=K_{c0}\exp(-\frac{E_{c}}{RT})$$

where *R* is the universal gas constant, and the global activation energies in Table 3 are used to calculate the chemical reaction rate constants.

The partial pressure $\alpha_{i}$ in Equation (1) is equal to the activity coefficient multiplied by the partial pressure of the component [38]:

(6) $$\alpha_{i}=\gamma_{i}x_{i}p$$

where $\gamma_{i}$ and $x_{i}$ are the activity coefficients and molar fractions of the component *i*, and *p* is the total reaction pressure in kPa.

The activity coefficients in Equation (6) are calculated by the following Equation [31]:

(7) $$RT\ln\gamma_{i}=\frac{(\beta_{i}+D_{i})p}{RT}$$

where $\beta_{i}$ and $D_{i}$ are given by:

(8) $$\beta_{i}=RTB_{i}-A_{i}-\frac{RC_{i}}{T^{2}}$$

(9) Di=(Ai12−∑ixiAi12)2+(Ci12−∑ixiCi12)2RT2−34RT(Bi13−∑ixiBi13)(Bi23−∑ixiBi23)

When the temperature is 653 K and the pressure is 15.6 MPa, each component’s partial pressure calculated by molar fraction and actual gas state are listed in Table 4. The overall difference between the results of the two calculations is minimal, but partial pressures calculated by the actual gas equation of state and activity are more accurate.

#### 2.1.2. Conservation Equation

Since the ammonia synthesis reactor is adiabatic, all the reaction heat is absorbed by the gas mixture inside the reactor. So the energy conservation equation in each axial micro-element can be represented by the change in temperature of the reacting gas [39,40]:

(10) $$\frac{dT}{dz}=\frac{A_{c}\rho_{c}\left(1-\varepsilon_{p}\right)\sum_{j}\eta_{j}r_{j}\Delta_{r}H_{j}}{\sum_{k}F_{k}C_{p,k}}$$

where $A_{c}$ is the cross-sectional area of the reactor in $m^{2}$; $\Delta_{r}H_{j}$ is the reaction enthalpy of reaction *j* in k·J/mol; $\eta_{j}$ is the effective factor of internal diffusion; $r_{j}$ is the intrinsic reaction rate of reaction j in mol/s;$\;c_{p,k}$ is the molar constant pressure heat capacity of component k in $J/(\mathrm{kg}\cdot K);F_{k}$ is the molar flow rate of component k in mol/s.

The mass conservation equation is expressed by the differential equation for the rate of conversion as follows:

(11) $$\frac{d\xi_{j}}{dz}=\frac{A_{c}\rho_{c}}{F_{k,\mathrm{in}}}\underset{j}{\sum }\eta_{j}r_{j}$$

After trial calculations, the Reynolds number of the gas mixture in the reactor ranges from 3000 to 12,000 in the given range of operating parameters, so the momentum conservation in the reactor is characterized by using the Hick’s pressure drop equation as follows [36,41]:

(12) dpdz=−6.8(1−εp)1.2εp3Re−0.2ρmvm2dp

#### 2.1.3. Entropy Generation Rate

The irreversibility losses for irreversible processes can be measured by entropy generation. Entropy generation in the reactor is mainly from the finite rate chemical reaction and the finite pressure-difference fluid flow, so the total entropy generation rate of the microelement length in the reactor is [42,43]:

(13) $$\sigma_{R}=A_{c}(1-\varepsilon_{p})\rho_{c}r\frac{\Delta G}{T}+A_{c}c_{g}\frac{1}{T}\frac{dp}{dz}$$

The total entropy generation rate of the whole reactor is the integral of the total entropy generation rate of the microelement length along the tube length.

(14) $$S_{\mathrm{GR}}=\int_{0}^{L}\sigma_{R}dz$$

### 2.2. Physical Model of Heat Exchangers

**Figure 3:**
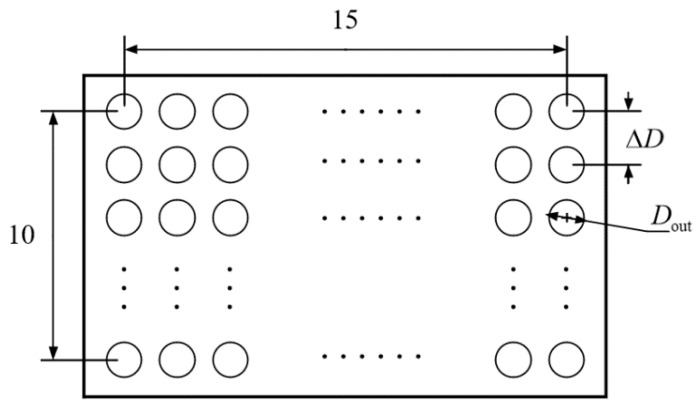
Cross section of counter flow heat exchanger.

The heat exchanger adopts counter-flow tube bundle heat transfer, with the high-temperature gas mixture from the reactor in the tube process and the water vapor at an initial pressure of 150 kPa and an initial temperature of 150 °C in the shell process. The schematic image of the cooler is shown in Figure 3, and its design parameters is in Table 5.

The Nusselt number for the heat transfer in the tube flow is calculated using the Gnielinski formula [44]:

(15) Nu=(f8)(Re−1000)Pr1+12.7f8(Pr23−1)[1+(Dinl)23](TfTw)0.45

The f in the Gnielinski formula is the Darcy resistance coefficient for turbulent flow in the tube, calculated from the Filonenko formula [44]:

(16) f=(1.82log10Re−1.64)−2

The same Gnielinski formula is used to express the heat transfer in the shell process flow, but the inner diameter of the pipe in Equation (15) needs to be changed to 4 times the hydraulic radius when calculating the Nu of the shell process.

(17) $$d=\frac{4A_{F}}{\chi }$$

where $A_{F}$ is the over-flow area in $m^{2}$; χ is the wetted circumference in m.

The pressure drop in the heat exchanger comes from the viscous frictional flow process and is calculated using Darcy’s equation [45] for the pressure drop along the flow, i.e.,

(18) $$\frac{dp_{H}}{dl_{H}}=f\frac{l_{H}}{d}\frac{c_{g}}{2g}$$

The heat transfer coefficients and heat transfer thermal resistance of the tube and shell processes can be obtained by the defined equation of Nu.

The total entropy generation rate in the cooler comes from the finite temperature difference heat transfer and the finite pressure difference flow processes, so the total entropy generation rate of the microelement length is given by [42,43]

(19) $$\sigma_{H}=\pi \cdot d_{r,\mathrm{in}}J_{q}\cdot (\frac{1}{T_{\mathrm{water}}}-\frac{1}{T_{\mathrm{mix}}})+A_{H}c_{g}\frac{1}{T}\frac{dp_{H}}{dz}$$

## 3. Optimization Methods

**Figure 4:**
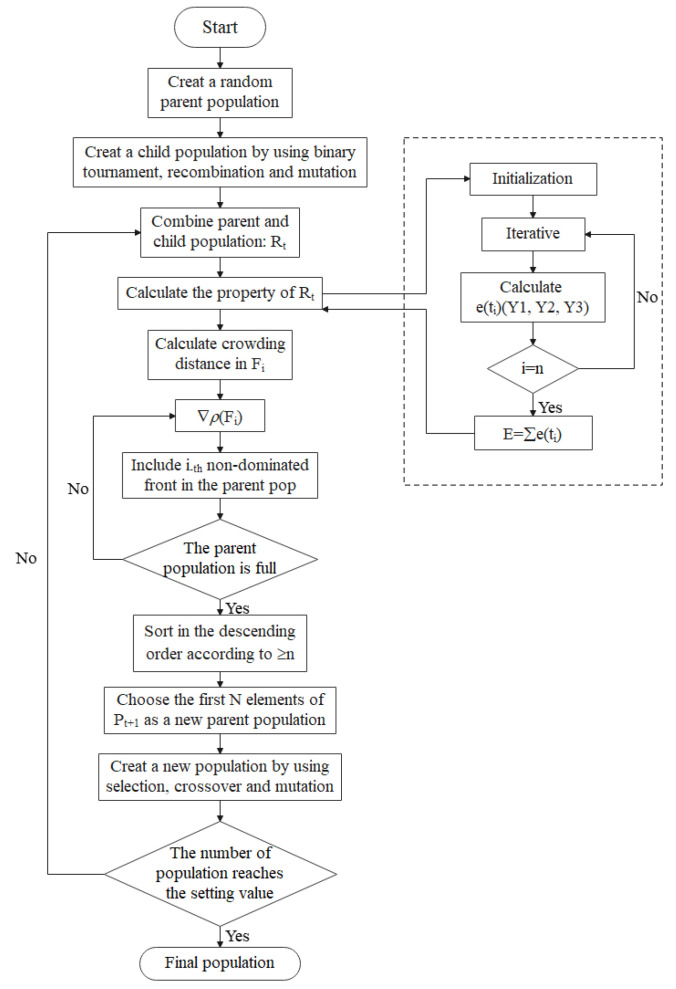
Algorithm flow chart of NSGA-II.

The application of industrial reactors to thermal storage and power generation requires the analysis and optimal design of its structural and operation parameters. In this paper, we analyzed the effects of eight single variables on the total exothermic rate and system entropy generation rate, and the variables includes the inlet molar flow rate, inlet gas hydrogen to nitrogen ratio, three reactor lengths and three reactor inlet temperatures. After that, considering minimum entropy generation rate and maximum exothermic rate, a non-dominated solution ranking genetic algorithm with elite strategy [46,47] (NSGA-II) will be used to optimize a total of seven variables simultaneously, including the inlet molar flow rate, three reactor lengths and three inlet temperatures. Figure 4 is the algorithm flow chart of NSGA-II [48].

Automatic machine learning algorithms [49,50,51,52,53,54,55,56,57,58] have been widely used in analysis and optimization in physical and chemical engineering. Liu et al. [51,52,54] used Bayesian optimization [51,52] and deep neural network algorithms [54] in the modelling and analysis of multiphase flow and boiling heat transfer, respectively. They effectively reduced the uncertainty of empirical correlations in complex processes and verified the effectiveness of the algorithms through experiments. For complex uncertain processes, machine learning algorithms have significant advantages on efficiency and accuracy.

All the flow processes of the model in this paper are single-phase, and the correlation formula can effectively express the reaction, flow and heat exchange processes. Confronted with this situation, the NSGA-II algorithm shows obvious advantages in multi-objective optimization. Firstly, the NSGA-II algorithm proposes a fast non-dominated sorting method, which effectively improves the speed of the search. Secondly, the concept of crowding is introduced in the algorithm, which ensures population diversity and effectively avoids falling into local optima. Finally, the inclusion of an elite strategy, which retains the outstanding parent population, can rapidly improve the quality of the population.

### 3.1. Univariate Analysis

The inlet molar flow rate is a key parameter affecting the total exothermic rate, and usually a larger inlet flow rate will lead to a higher total exothermic rate. Considering the reference reactors inlet flow rate is 132 mol/s, a more suitable molar flow rate for the exothermic scenario is explored in the range of 110–180 mol/s.

The effect of gas hydrogen to nitrogen ratio($R_{H,N}$) on performance is analyzed by controlling the inlet molar flow rate to be constant. Since the stoichiometric ratio of hydrogen to nitrogen in the reaction equation is 3, the hydrogen to nitrogen ratio is continuously varied from 2 to 4 to find a better performing hydrogen to nitrogen ratio.

When the effect of individual reactor length on performance is analyzed, because the length of the reactor 3 is almost twice as long as the reactors 1 and 2, the analysis range for the reactors 1 and 2 is 0.5 m above and below their reference values and the analysis range for the reactor 3 is 1 m above and below the reference value.

When the effect of individual reactor inlet temperature on performance is analyzed, the temperature analysis range for each reactor is 50 K above and below its reference value.

### 3.2. Multivariate Optimization

The NSGA-II algorithm is one of the most applied multi-objective optimization algorithms, which introduces an elite strategy to ensure that the good individuals of the parent generation are not discarded to improve the optimization accuracy.

Through univariate analysis, the influence of each structural or operation parameter of the reactor on the overall performance is investigated, and combining with the parameters of the reference reactors, the parameter interval of multi-objective optimization is clarified. Finally, with the minimum entropy generation rate and maximum exothermic rate as objectives, a total of seven variables, including inlet molar flow rate, lengths and inlet temperatures of the three reactors, are simultaneously optimized to obtain the Pareto fronts. The objective function and constraints of the optimization problem are as follows:

(20) $$\min(-Q,S_{G})$$

(21) $$s.t.\left\{ \begin{array}{l} 110\;\mathrm{mol}/s<N<180\;\mathrm{mol}/s\\ 3.2\;m<L_{1}<3.8\;m\\ 3.7\;m<L_{2}<4.3\;m\\ 6.2\;m<L_{3}<6.8\;m\\ 620\;K<T_{1}<680\;K\\ 630\;K<T_{2}<690\;K\\ 630\;K<T_{3}<690\;K \end{array}\right.$$

## 4. Numerical Example of Univariate Analysis

### 4.1. Model Validation

The model calculation results obtained at an inlet flow rate of 132 mol/s and the actual engineering results [59,60] are shown in Table 6. The comparison shows that the deviation of the model calculation in this paper is from −3.04% to 17.82%, and the deviation of the final node is only 7.76%. The errors mainly come from that the one-dimensional model ignores the effect of gravity, whereas the actual Braun ammonia synthesis tower is vertical. At the same time, a maximum temperature deviation of 3.04% and a final ammonia molar fraction deviation of 7.76% are acceptable as the paper is concerned with the overall exothermic rate and entropy generation rate. This indicates that this model is accurate and can effectively simulate the exothermic rate of reactor.

In industry, coke, water vapor and air are used to make reaction gases. Multiple cycles result in an increased content of rare gases in the gas mixture, mainly argon. When this reactor is applied to an ammonia-based thermochemical heat storage system, its inlet reaction gas comes from the front ammonia decomposition heat storage reactor, so it can be assumed that the inlet gas composition contains only hydrogen, nitrogen and ammonia gas. Therefore, the molar fraction of each component of the inlet gas of the reference reactor needs to be adjusted to the data in Table 7.

### 4.2. Effect of Inlet Molar Flow Rate on System Performance

**Figure 5:**
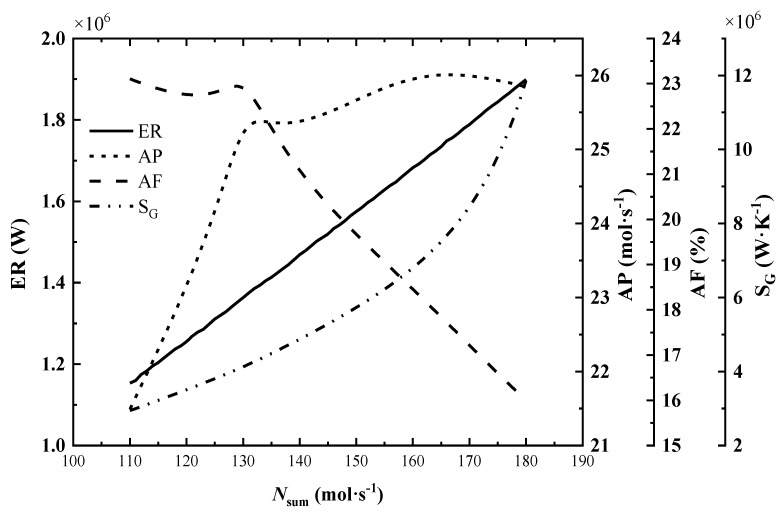
Effect of inlet molar flow rate on system performance.

The variation of total exothermic rate (ER), total entropy generation rate (S_G_), ammonia production rate (AP) and exit ammonia molar fraction (AF) with the increase in inlet molar flow rate is shown in Figure 5.

As seen in Figure 5, the total exothermic rate increases almost linearly and steadily with the increase in inlet molar flow rate, while the total entropy generation rate climbs sharply. By observing the outlet parameters of each reactor under different flow rates, it is found that the ammonia production rate and exothermic rate increase with the increase in inlet flow rate, but the molar fraction of ammonia and the reactor outlet temperature decrease. This indicates that the reaction is far from the equilibrium state and is more favorable for the advance of positive reaction to improve the exothermic rate continuously. The dramatic increase in the total entropy generation rate is mainly due to the rise of the inlet flow rate and the ammonia synthesis rate, which leads to a significant increase in the entropy generation rate of the flow process and chemical reaction.

In Figure 5, when the inlet molar flow rate is below 130 mol/s, the ammonia production rate increases continuously with the increase in inlet flow rate, and the change of exit ammonia molar fraction is not obvious; after the inlet flow rate exceeds 130 mol/s, the ammonia production rate increases slowly and the exit ammonia molar fraction decreases rapidly.

Therefore, when the inlet molar flow rate is 132 mol/s, the reference value, the reactor has a high AP and AF. This is more in line with the needs of industrial ammonia synthesis. While, for the exothermic reactor, it’s necessary to optimize the inlet molar flow rate.

### 4.3. Effect of Hydrogen to Nitrogen Ratio on System Performance

The effect of the hydrogen–nitrogen ratio on the system performance is analyzed by increasing the hydrogen-nitrogen ratio($R_{H,N}$) from 2 to 4 at constant inlet flow rates of 120, 130 and up to 180 mol/s, respectively.

**Figure 6:**
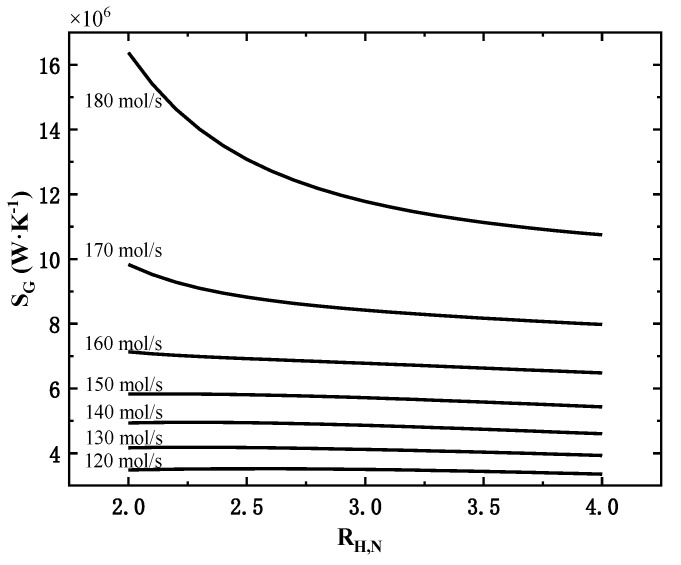
Effect of hydrogen–nitrogen ratio on the total entropy generation rate at different molar flow rates.

Under the constant molar flow rate, the effect of inlet gas hydrogen–nitrogen ratio on the total exothermic rate is not obvious (the maximum change is 1%), and the total exothermic rate shows a slight decrease with the increase in hydrogen-nitrogen ratio. The variation of the total entropy generation rate of the system with the hydrogen to nitrogen ratio is shown in Figure 6.

As shown in Figure 6, under different molar flow rates, the variation of total entropy generation rate is different. When the inlet flow rate is 150 mol/s and below, the total entropy generation rate increases slightly and then decreases slowly; when the inlet flow rate is 160 mol/s and above, the total entropy generation rate decreases continuously with the increase in hydrogen-nitrogen ratio, and the larger the inlet flow rate, the more significant this decrease is. So, when the inlet flow rate is large, the hydrogen to nitrogen ratio should be increased as much as possible to effectively reduce the total entropy generation rate with little effect on the total exothermic rate.

The main reason for the above-mentioned rules is that when the inlet flow rate is large and the hydrogen–nitrogen ratio is low (more nitrogen content), the remaining nitrogen content is still high after entering the third tower. The high nitrogen content can effectively promote the ammonia synthesis reaction, making the third reaction tower’s outlet temperature and total entropy generation rate much higher.

### 4.4. Effect of Each Reactor’s Length on System Performance

**Figure 7:**
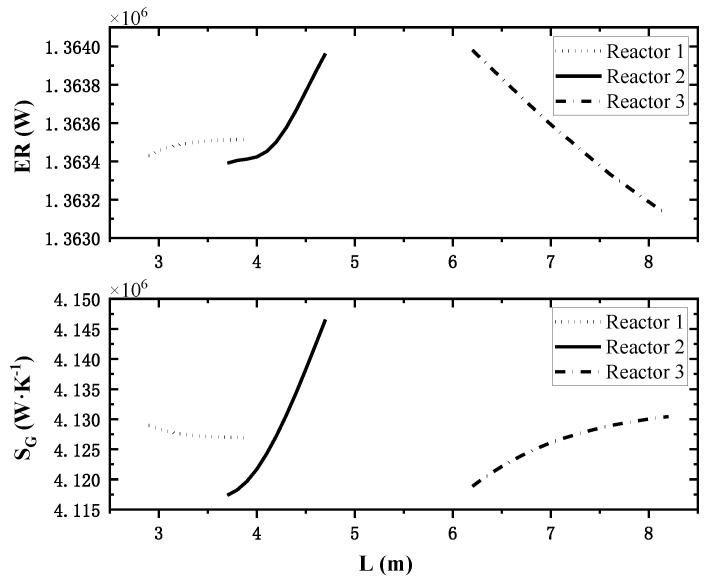
Effect of single reactor’s length on system performance.

In this paper, three reactor towers are involved, so the effect of the variation of the third reactor length on the performance index is analyzed when the other two reactor’s lengths are maintained at reference values. The changes of total exothermic rate and total entropy generation rate corresponding to the variation of each reactor’s length are shown in Figure 7.

The different lines in Figure 7 represent the length change process of different reactors, and the comparison between the top and bottom shows that: in the calculation range, as the length of reactor 1 increases, the total heat release rate increases and the total entropy generation rate decreases, and the change process tends to be smooth, so reactor 1 should be longer within a reasonable range. The increase in reactor 2 length will incease the total heat release rate and the total entropy generation rate simultaneously. Therefore, it is necessary for reactor 2 to coordinate between the two indexes to obtain a better length. As the length of reactor 3 increases, the total heat release rate decreases and the total entropy generation rate increases, so reactor 3 should be as short as possible.

### 4.5. Effect of Each Reactor’s Inlet Temperature on System Performance

**Figure 8:**
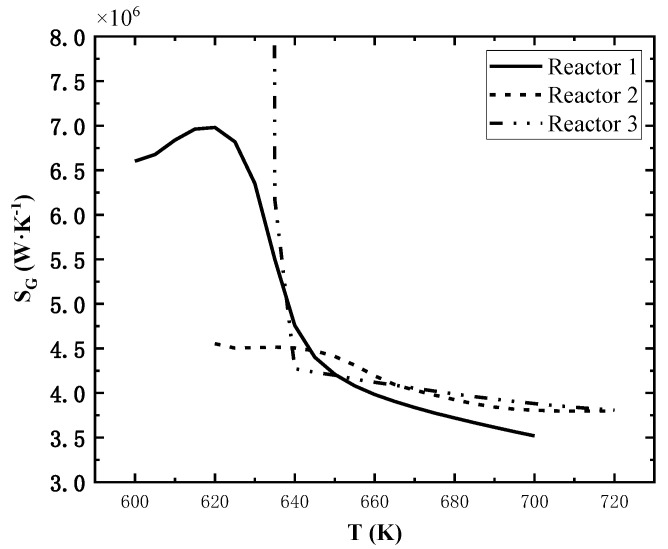
Effect of single reactor’s inlet temperature on total entropy generation rate.

Similarly, two reactors’ inlet temperatures are controlled as reference values, and the third reactor inlet temperature varies in the range of 50 K above and below the reference value. The effect of individual reactor inlet temperature variations on the total entropy generation rate is shown in Figure 8.

The effect of inlet temperature on the total exothermic rate is also not obvious, but that on total entropy generation rate is significant. As shown in Figure 8, the total entropy generation rate increases firstly and then decreases rapidly as the inlet temperature of the reactor 1 increases; the total entropy generation rate decreases continuously as the inlet temperature of the reactor 2 increases; for the reactor 3, the total entropy generation rate is extremely high when the inlet temperature is lower than 635 K and decreases sharply as the inlet temperature increases, and it decreases slowly when the inlet temperature is much higher than 635 K.

## 5. Numerical Example of Multivariate Optimization

**Figure 9:**
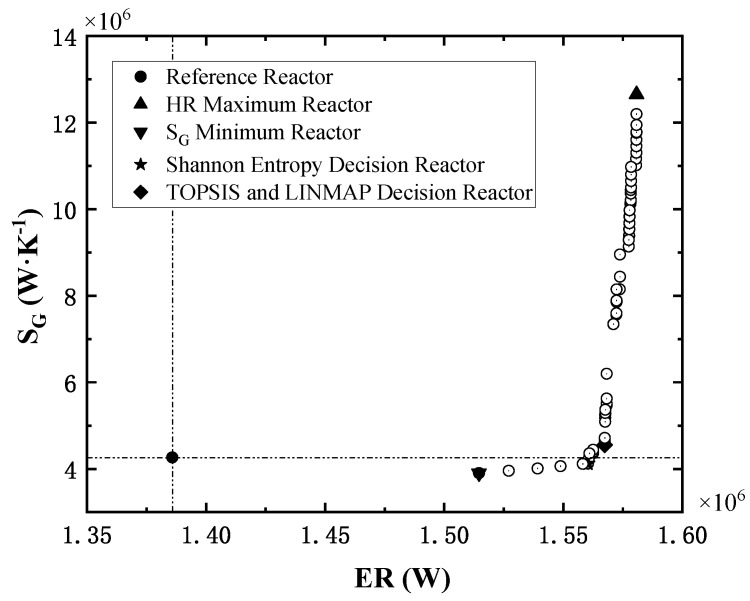
The Pareto front of multi-objective optimization.

Taking minimum entropy generation rate and maximum exothermic rate as objectives, the NSGA-II algorithm [43] is applied to fulfill the multi-objective optimization. The Pareto front obtained is shown in Figure 9.

In Figure 8, the solid dot in the lower left corner is the performance index of the reference reactor, and the reference point will divide the Pareto front space in quadrants. The points of Pareto front all locate in the first and fourth quadrants and show a tendency to converge to the lower right corner (ideal point), indicating that the optimal reactors significantly enhance the total exothermic rate. Compared with the reference reactor, the points in the fourth quadrant reduce the entropy generation rate while enhancing the exothermic rate.

Two sets of structural and operation parameters are selected from the Pareto front by three fuzzy decision methods, i.e., TOPSIS [61,62,63], LINMAP [64,65] and Shannon Entropy [66,67,68]. The optimal reactors determined by different decision methods are labeled in Figure 9, and the related parameters are listed in Table 8. Finally, based on the deviation index (DI) of the two decision points, the Shannon Entropy decision reactor is chosen as the optimized reactor. The DI is the ratio of the distance of the decision point from the optimized solution to the sum of the distance of the decision point from the optimized solution and the worst solution. The smaller the DI, the better the results.

(22) $$DI=\frac{D\mathrm{ideal}}{D\mathrm{idela}+D\mathrm{non}}$$

(23) $$D\mathrm{ideal}=\sqrt{{\left(Sx-S\min\right)}^{2}+{\left(Qx-Q\max\right)}^{2}}$$

(24) $$D\mathrm{non}=\sqrt{{\left(Sx-S\max\right)}^{2}+{\left(Qx-Q\min\right)}^{2}}$$

The *D*_ideal_ is the distance from the decision point to the ideal point and the *D*_non_ is the distance from the decision point to the non-ideal point.

## 6. Conclusions

In this paper, a one-dimensional model of Braun-type ammonia synthesis exothermic reactor is established based on finite-time thermodynamics, and the effects of parameters such as inlet molar flow rate, hydrogen-nitrogen ratio, reactor length, and reactor inlet temperature on system performance are analyzed. The Pareto front is obtained by NSGA-II algorithm, and three decision methods including TOPSIS, LINMAP and Shannon Entropy, are applied to obtain optimal reactors. The analysis and optimization results show that:

1. The inlet molar flow rate is the key parameter affecting the total exothermic rate of the system. The total exothermic rate basically increases linearly with the increase in the molar flow rate, but the total entropy generation rate climbs sharply.
2. Under the constant inlet molar flow rate, the change of hydrogen to nitrogen ratio has little effect on the total exothermic rate. But when the molar flow rate is large, the hydrogen to nitrogen ratio has an obvious effect on the total entropy generation rate, and a higher hydrogen to nitrogen ratio should be chosen for large molar flow rate.
3. Compared with the reference reactor, the TOPSIS and LINMAP optimal reactor improves the total exothermic rate by 13.1% and the total entropy generation rate by 6.7%; the Shannon entropy optimal reactor improves the total exothermic rate by 12.6% and reduces the total entropy generation rate by 3.4%.
4. According to the deviation index, the Shannon Entropy optimal reactor is choosen as the optimized reactor.

## Figures and Tables

**Table 1:** Design and operating parameters for reference reactors.

Parameters	Symbol	Value
Inner diameter of reactor 1	$D_{1}$	2.46 m
Inner diameter of reactor 2	$D_{2}$	2.82 m
Inner diameter of reactor 3	$D_{3}$	2.82 m
Inlet temperature of reactor 1	$T_{1}$	653 K
Inlet temperature of reactor 2	$T_{2}$	663 K
Inlet temperature of reactor 3	$T_{3}$	659 K
Length of reactor 1	$L_{1}$	3.3 m
Length of reactor 2	$L_{2}$	4.2 m
Length of reactor 3	$L_{3}$	7.2 m
Reaction pressure	p	15 MPa
Catalyst particle diameter	$D_{c}$	0.007 m
Porosity of catalyst bed	$\varepsilon_{p}$	0.6
Molar fraction of hydrogen	$R_{H}$	0.7
Molar fraction of nitrogen	$R_{N}$	0.23
Molar fraction of ammonia	$R_{Z}$	0.035
Molar fraction of argon	$R_{A}$	0.035
Molar flow rate of inlet gas	$N_{\mathrm{sum}}$	132 mol/s

**Table 2:** Parameters in Beattie–Bridgeman Equation [31].

Substance	*i*	$\;A_{i}(10^{-3}{J\cdot m}^{3}/{\mathrm{mol}}^{2})$	$\;\;B_{i}(10^{-6}m^{3}/\mathrm{mol})$	$\;C_{i}(K^{3}\cdot m^{3}/\mathrm{mol})$
Hydrogen	H	20.01	20.96	0.504
Nitrogen	N	136.23	50.46	4768.7
Ammonia	Z	242.47	34.15	59.9
Argon	A	130.78	39.31	128.3

**Table 3:** Parameters of Equations (4) and (5) [31].

*K*_b0_ $$\begin{array}{l} \mathrm{mol}\cdot \mathrm{atm}/\left(m^{3}\cdot s\right) \end{array}$$	*E*_b_ $$\begin{array}{l} k\cdot J/\mathrm{mol} \end{array}$$	*K*_c0_ atm12	*E*_c_ $$\begin{array}{l} k\cdot J/\mathrm{mol} \end{array}$$
2.19 × 10^10^	46.752	2.94 × 10^−4^	−100.66

**Table 4:** Comparison of partial pressure.

Components	Hydrogen	Nitrogen	Ammonia
Molar fraction	0.7	0.25	0.05
Partial pressure calculated by molar fraction (bar)	109.2	39.0	7.8
Partial pressure calculated by actual gas state (bar)	109.1	39.0	7.9
Difference (bar)	0.1	0	−0.1

**Table 5:** The model parameters of heat exchanger.

Parameters	Symbol	Value
Inner diameter of tube	$D_{\mathrm{in}}$	0.05 m
Outer diameter of tube	$D_{\mathrm{out}}$	0.047 m
Thermal conductivity of tube wall	$k_{T}$	$21.5\;W/{(K\cdot m}^{2})$
Axis distance between tubes	ΔD	0.08 m
Number of tubes	n	15 × 10

**Table 6:** Comparison of model results with actual results.

Parameters	Reference Reactor [59,60]	Model	Deviation
Outlet temperature of reactor 1	784 K	783.6 K	−0.05%
Outlet temperature of reactor 2	740 K	717.5 K	−3.04%
Outlet temperature of reactor 3	712 K	708.3 K	−0.52%
Outlet molar fraction of ammonia of reactor 1	11.67%	13.75%	17.82%
Outlet molar fraction of ammonia of reactor 2	16.84%	18.27%	8.49%
Outlet molar fraction of ammonia of reactor 3	21.01%	22.64%	7.76%

**Table 7:** Components of inlet gas mixture.

	Hydrogen	Nitrogen	Ammonia
Reference value	0.7	0.23	0.035
Modified value	0.72	0.24	0.04

**Table 8:** Decision reactors’ parameters in multi-objective optimization.

Decision Reactor	*N* (mol/s)	$L_{1}$ (m)	$L_{2}$ (m)	$L_{3}$ (m)	$T_{1}$ (K)	$T_{2}$ (K)	$T_{3}$ (K)	Q (kW)	S_G_ (kW/K)	DI
Reference reactor	132	3.3	4.2	7.2	653	663	659	1385.8	4265.6	
Exothermic rate maximizing reactor	148	3.4	4.3	6.8	620	630	630	1580.7	12,650.8	
Entropy generation minimizing reactor	144	3.4	4.3	6.2	680	690	690	1514.6	3903.1	
TOPSIS and LINMAP reactor	148	3.8	3.7	6.2	680	630	690	1567.3	4552.7	0.1231
Shannon Entropy reactor	148	3.8	3.7	6.2	680	690	690	1560.4	4121.5	0.0315

## Data Availability

Not applicable.

## Nomenclature

$A_{c}$	Cross-section area, $m^{2}$
$C_{p}$	Molar heat capacity, $J\cdot {\mathrm{mol}}^{-1}\cdot K^{-1}$
*D*	Diameter, m
$N_{\mathrm{sum}}$	Molar flow rate, $\mathrm{mol}\cdot s^{-1}$
*K*_p_	Equilibrium constant
L	Length, m
M	Molar mass, ${\mathrm{kg}\cdot \mathrm{mol}}^{-1}$
Pr	Prandtl number
p	Reaction pressure, Pa
R	Universal gas constant, ${J\cdot \mathrm{mol}\cdot K}^{-1}$
$R_{i}$	Molar fraction of component *i*
Re	Reynolds number
r	Chemical reaction rate, ${\mathrm{mol}\cdot s}^{-1}\;$
*S*_G_	Entropy generation rate, ${J\cdot K}^{-1}{\cdot \mathrm{mol}}^{-1}$
T	Temperature, K
α	Activity coefficient of partial pressure
ε	Porosity
μ	Dynamic viscosity, ${\mathrm{kg}\cdot m}^{-1}{\cdot s}^{-1}$
σ	Local entropy generate rate, ${J\cdot K}^{-1}{\cdot m}^{-1}{\cdot s}^{-1}$
ρ	Density, $\mathrm{kg}\cdot m^{-3}$
λ	Covariate variable vector
γ	activity coefficient
ΔG	Gibbs free energy change of reaction, ${J\cdot \mathrm{mol}}^{-1}$
ΔH	Enthalpy of reaction, ${J\cdot \mathrm{mol}}^{-1}$
AP	Ammonia production rate
AF	Exit ammonia molar fraction
ER	Total exothermic rate
